# A Novel Two-Dimensional TiClO as a High-Performance Anode Material for Mg-Ion Batteries: A First-Principles Study

**DOI:** 10.3390/ma16103876

**Published:** 2023-05-21

**Authors:** Songcheng Zhang, Chunsheng Liu

**Affiliations:** College of Electronic and Optical Engineering, Nanjing University of Posts and Telecommunications, Nanjing 210023, China; 1220024804@njupt.edu.cn

**Keywords:** magnesium-ion batteries, 2D materials, TiClO, anode, first-principles calculations

## Abstract

Searching for efficient electrode materials with excellent electrochemical performance is of great significance to the development of magnesium-ion batteries (MIBs). Two-dimensional Ti-based materials are appealing for use in MIBs due to their high cycling capability. On the basis of density functional theory (DFT) calculations, we comprehensively investigate a novel two-dimensional Ti-based material, namely, TiClO monolayer, as a promising anode for MIBs. Monolayer TiClO can be exfoliated from its experimentally known bulk crystal with a moderate cleavage energy of 1.13 J/m^2^. It exhibits intrinsically metallic properties with good energetical, dynamical, mechanical, and thermal stabilities. Remarkably, TiClO monolayer possesses an ultra-high storage capacity (1079 mA h g^−1^), a low energy barrier (0.41–0.68 eV), and a suitable average open-circuit voltage (0.96 V). The lattice expansion for the TiClO monolayer is slight (<4.3%) during the Mg-ion intercalation. Moreover, bilayer and trilayer TiClO can considerably enhance the Mg binding strength and maintain the quasi-one-dimensional diffusion feature compared with monolayer TiClO. All these properties indicate that TiClO monolayers can be utilized as high-performance anodes for MIBs.

## 1. Introduction

Lithium-ion batteries (LIBs) have dominated the portable device industry and the electric vehicle market in the last 30 years due to their high energy density and repeated cycling stability [1,2]. However, the shortage and uneven geographical distribution of lithium resources significantly affect the large-scale application of LIBs [3,4,5]. Rechargeable MIBs have been regarded as a potential alternative to LIBs because of their abundant resources, intrinsic nontoxicity, and high theoretical capacity [6,7,8,9]. However, the research on MIBs is still in its infancy, and several serious problems need to be resolved. One of the most critical challenges is the irreversible formation of a passivation layer on the surface of the Mg anode in most electrolyte species, resulting in sluggish diffusion of Mg ions [10]. Therefore, identifying novel anode materials as alternatives to Mg metal would be an effective method to overcome this obstacle.

Since the synthesis of graphene by Geim et al. [11,12] in 2004, two-dimensional (2D) materials have captured a great deal of research interest in the field of electrochemistry [13]. As compared to bulk materials, 2D materials possess huge surface areas and dense ion insertion positions, which make them one of the most optimal choices for electrode materials. However, only a few synthesized 2D materials can be utilized as anodes for high-performance MIBs. For instance, WS_2_ monolayer, as one of the transition metal disulfides, has been extensively explored as an electrode material for ion battery applications. It exhibits a high storage capacity (361 mA h g^−1^) when used as an anode material for MIBs [14]. However, the inferior conductivity of pristine WS_2_ seriously restricts its rate and cycling capability. Phosphorene, which is exfoliated from layered black phosphorus, shows a high Mg capacity (865 mA h g^−1^) and a rather low Mg diffusion barrier (0.09 eV) along the zigzag direction [15,16]. However, phosphorene cannot maintain its structure in ambient conditions, which may significantly influence its feasibility in practical application [17].

Ti-based oxides have been considered to be the most promising Mg^2+^ insertion-type anode materials because of their nontoxicity, resource abundance, and remarkable cycling capability [18,19,20]. TiO_2_, as one of the typical Ti-based oxides, has tremendous electrochemical performance as an anode for Li/Na-ion batteries owing to its excellent cycle stability [21,22]. Beyond that, TiOF_2_ nanostructure is revealed theoretically to be a good candidate as an anode material for LIBs with high specific capacity (1045 mA h g^−1^) [23]. Recently, several layered ternary Ti-based oxides (e.g., TiOF, TiClO) have been synthesized with metallic behaviors. TiOF monolayer, which can be obtained via exfoliation from its bulk counterpart, has been investigated as an anode material for LIBs. It possesses an excellent capacity (970 mA h g^−1^) and a superior diffusion barrier (0.15 eV) [24]. Nevertheless, the electrochemical performance of the TiClO monolayer in MIBs has yet to be studied.

In this work, we present a comprehensive theoretical study of a novel 2D TiClO monolayer using first-principles calculations. We first investigate the structural and electronic properties of TiClO monolayers. The results show that the TiClO monolayer is mechanically, dynamically, and thermally stable with metallic characteristics. Then, we study the adsorption and diffusion of Mg atoms on a TiClO monolayer to assess its feasibility as an anode for MIBs. Our calculations reveal that TiClO monolayer possesses a specific capacity of 1079 mA h g^−1^, which is higher than those of some reported 2D anode materials. Furthermore, it also exhibits a low diffusion barrier (0.41–0.68 eV) and a small lattice change (<4.3%) during the Mg ion intercalation process. Finally, we explore the Mg adsorption property of bilayer TiClO.

## 2. Computational Methods

All spin-polarized density functional theory (DFT) calculations are carried out using the Cambridge sequential total energy package (CASTEP) [25]. The norm-conserving pseudopotentials are carried out to describe the electron-ion interaction [26]. The plane-wave cut-off energy is set to 1100 eV for all the calculations. A vacuum space of 20 Å is constructed to minimize the virtual interlayer interactions under the periodic boundary condition. The generalized gradient approximation (GGA) of the Perdew–Burke–Ernzerhof (PBE) functional is used as the exchange-correlation functional [27]. The long-range van der Waals (vdW) forces are considered via the DFT-D3 method with Grimme correction [28]. The hybrid Heyd–Scuseria–Ernzerhof (HSE06) hybrid functional is adopted to obtain an accurate band structure [29]. The Brillouin zone (BZ) is sampled with a 30 × 26 × 1 Monkhorst-Pack *k*-grid [30]. The two-point steepest descent (TPSD) [31] is implemented in the geometry optimizations, in which the convergence threshold is set as 5 × 10^−7^ eV/atom in energy and 10^−3^ eV/Å in force. The phonon band dispersions are calculated with the finite displacement method [32]. The thermal stability is assessed by carrying out ab initio molecular dynamics (AIMD) simulations [33] in the NVT ensemble in a supercell of 4 × 4 × 1 at 600 K. The total simulation lasts 10 ps, with a time step of 1 fs. The climbed image nudged elastic band (CI-NEB) method is employed to investigate the diffusion energy barrier for the migration of Mg atoms on TiClO [34].

## 3. Results and Discussion

### 3.1. Structure and Cleavage Energy of Bulk TiClO

**Figure 1:**
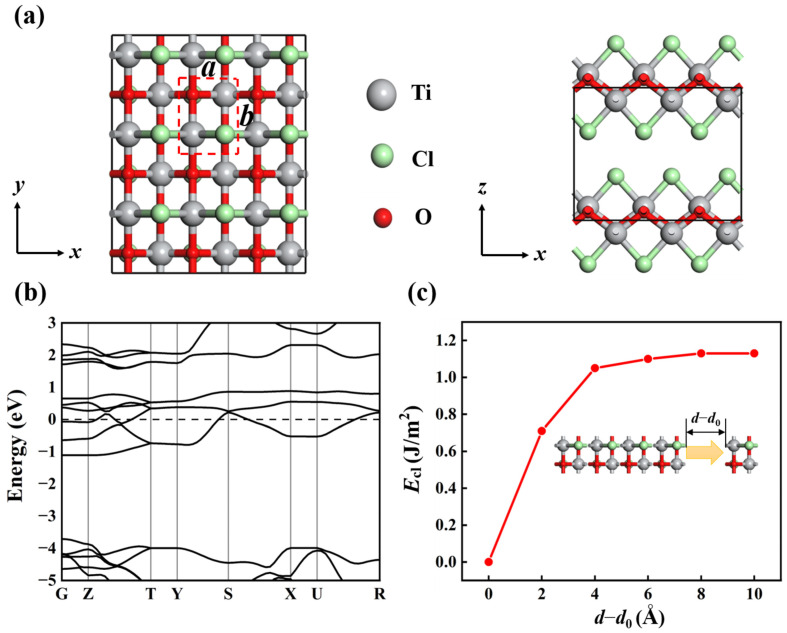
(**a**) Top and side views of bulk TiClO structure with 3 × 3 × 1 supercell. A unit cell is represented by the red dashed rectangle. (**b**) Electronic band structure of bulk TiClO calculated with PBE method. (**c**) Cleavage energy (*E*_cl_) as a function of the separation distance (*d*) between separated monolayer and its neighboring bulk structure, where *d*_0_ represents the interlayer distance between nearest-neighboring layers in bulk TiClO.

The bulk TiClO crystallizes in a layered structure of the FeOCl type, where buckled bilayers of Ti-O are separated by Cl ions, as shown in Figure 1a [35]. The unit cell is orthorhombic, with space group *Pmmn* (No. 59). The optimized lattice constants are *a* = 3.255 Å, *b* = 3.977 Å, and *c* = 7.716 Å, which are in good accordance with the experimental data (*a* = 3.365 Å, *b* = 3.789 Å, and *c* = 8.060 Å) [36]. In addition, bulk TiClO is metallic, with energy levels crossing the Fermi level (Figure 1b).

In general, liquid-phase exfoliation and mechanical cleavage are two main techniques for obtaining monolayer structures from their bulk counterparts. To assess the viability of exfoliating the TiClO monolayer from its layered bulk crystal, we calculate the cleavage energy (*E*_cl_) of the TiClO monolayer. To simulate the actual exfoliation process, we construct a five-layer slab model of TiClO in which only the top layer is removable. As shown in Figure 1c, the value of *E*_cl_ increases with the increase of the separation distance (*d*) and gradually converges to a constant number of ~1.13 J/m^2^ at about 10 Å. This value is comparable to those of some layered materials, such as Ca_2_N (1.09 J/m^2^) [37], GeP_3_ (1.14 J/m^2^) [38], and CaP_3_ (1.30 J/m^2^) [39], indicating the high feasibility of exfoliating the TiClO monolayer from its bulk crystal.

### 3.2. Structure and Stability of Monolayer TiClO

**Figure 2:**
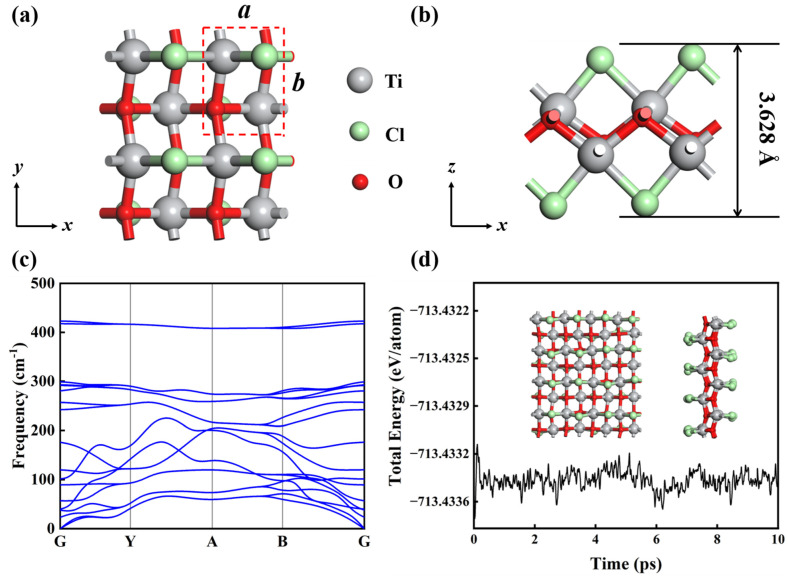
Top (**a**) and side (**b**) views of monolayer TiClO. A unit cell with lattice parameters is represented by red dashed lines. (**c**) Phonon spectra of TiClO. (**d**) Evolution of total energy profile during AIMD simulation at 600 K for 10 ps. The insets are snapshots of the last step.

The fully optimized geometrical structure of an exfoliated TiClO monolayer is presented in Figure 2a,b. The relaxed lattice constants are *a* = 3.303 Å and *b* = 3.833 Å, which are close to those of the bulk phase. As shown in Figure 2b, each TiClO monolayer is composed of six close-packed atomic layers sequenced Cl-Ti-O-O-Ti-Cl. Each Cl atom bonds with two Ti atoms, and each Ti atom bonds with two Cl atoms and four O atoms. The bond lengths of Ti-O and Ti-Cl are 2.058 and 2.476 Å, respectively.

The materials used as anodes for MIBs should have good energy, dynamical, mechanical, and thermal stability. Firstly, we calculate the cohesive energy ($E_{b}$) to examine the energy stability of TiClO monolayer, which can be described as:

(1) $$E_{b}={(xE}_{Ti}+{yE}_{Cl}+{zE}_{O}-E_{TiClO})/(x+y+z)$$

where $E_{Ti}$, $E_{Cl}$, and $E_{O}$ refer to the total energies of a single Ti, Cl, and O atom, respectively. The $E_{TiClO}$ denotes the total energy of monolayer TiClO. x, y and z are the number of Ti, Cl, and O atoms in the TiClO monolayer, respectively. The positive cohesive energy represents a structure that is stable. The $E_{b}$ of TiClO monolayer (7.42 eV/atom) is higher than that of 2D Ti_2_BN (4.90 eV/atom) [40] and comparable to that of 2D Ti_2_C (7.49 eV/atom) [41] calculated at the same theoretical level, denoting that the TiClO monolayer is energetically stable. We further evaluate the thermodynamic stability of TiClO monolayers by computing the formation energy ($E_{f}$) from the following Equation: $E_{f}={(E}_{Ti}+E_{Cl}+E_{O}-E_{TiClO})/3$. $E_{Ti}$, $E_{Cl}$ and $E_{O}$ are the total energies of Ti, Cl, and O atoms in bulk titanium, chlorine molecules, and oxygen molecules, respectively. $E_{TiClO}$ is the total energy of the TiClO monolayer. The calculated formation energy of the TiClO monolayer is 0.34 eV, confirming that the TiClO monolayer is thermodynamically stable.

Then, we compute the phonon spectra to investigate the dynamical stability. As shown in Figure 2c, no imaginary phonon modes are observed in the whole Brillouin zone, indicating the dynamic stability of TiClO. We further determine the mechanical stability by examining the elastic constants. The obtained elastic constants are $C_{11}$ = 106.93 N/m, $C_{12}$ = 21.20 N/m, $C_{22}$ = 108.47 N/m, and $C_{66}$ = 39.44 N/m, which satisfy the Born-Huang criteria ($C_{11}C_{22}-C_{12}^{2}\;>\;0$, $C_{66}\;>\;0$) [42], suggesting TiClO monolayer is mechanically stable. Moreover, we confirm the thermal stability of monolayer TiClO by performing AIMD simulations at a temperature of 600 K for the 4 × 4 × 1 supercell. As illustrated in Figure 2d, the TiClO monolayer maintains its structure without relevant distortions after heating for 10 ps, indicating that it is thermally stable.

### 3.3. Electronic Property of Monolayer TiClO

**Figure 3:**
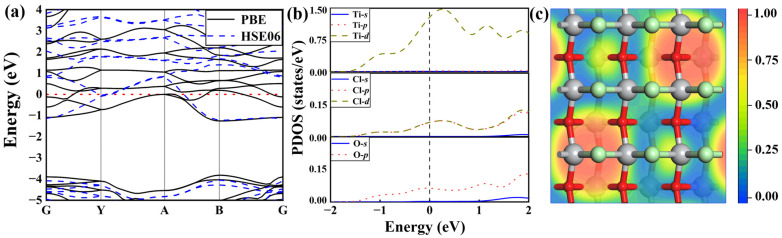
(**a**) Electronic band structures of the TiClO monolayer calculated with PBE and HSE06 functionals. (**b**) The partial DOS of the TiClO monolayer under the PBE method. (**c**) ELF map of the TiClO monolayer.

To better understand the electronic properties of TiClO monolayers, we calculate their band structure and orbital-projected density of states (DOS). As illustrated in Figure 3a, the TiClO monolayer possesses metallic characteristics as several energy levels cross the Fermi level with both PBE and HSE06 functionals, ensuring good electronic conductivity. The partial DOS suggests that the metallic states in the vicinity of Fermi level are predominantly contributed by Ti-*d* orbitals, while the O-*p*, Cl-*p,* and Cl-*d* orbitals only make a small contribution (Figure 3b). To further explore the mechanism of chemical bonding, we also compute the electron localization function (ELF) of the TiClO monolayer. As presented in Figure 3c, the ELF values (<0.5) of Ti-O and Ti-Cl bonds correspond to the ionic bond.

### 3.4. Mg Adsorption on Monolayer TiClO

**Figure 4:**
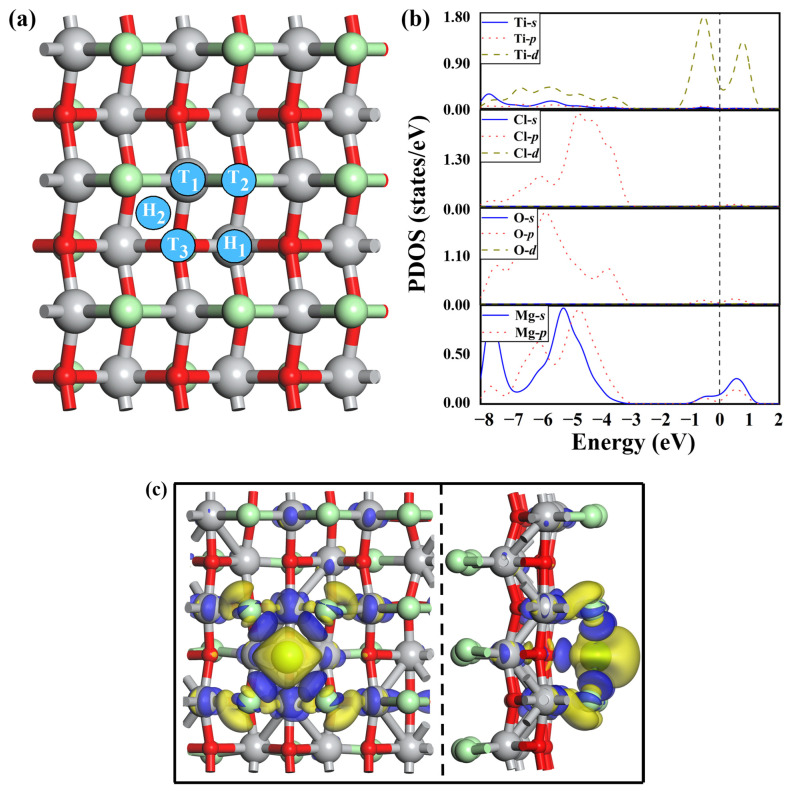
(**a**) Top view of the representative Mg adsorption sites on the surface of TiClO monolayer: top (T_1_, T_2_, and T_3_) and hollow (H_1_ and H_2_). (**b**) The partial DOS of a single Mg adsorbed on TiClO monolayer at site T_3_. (**c**) Top and side views of charge density difference plots for Mg adsorption on the T_3_ site of TiClO. The blue and yellow colors denote the gain and loss of electrons, respectively.

In order to explore the feasibility of a TiClO monolayer as an anode material for MIBs, we studied the adsorption of an isolated Mg atom on TiClO by constructing a 3 × 3 × 1 supercell. As shown in Figure 4a, five possible adsorption sites with high structural symmetry are chosen, denoted as T_1_, H_1_, H_2_, T_2_, and T_3_. During the structural relaxation, the adsorbed Mg atom at site H_2_ would move to the near site T_3_, and the Mg atom on site T_2_ would finally shift to the neighboring site T_1_. However, the Mg on site H_1_ stays still. The adsorption energy ($E_{ads}$) of a Mg atom on a TiClO monolayer is defined as:

(2) $$E_{ads}=E_{Mg}+E_{TiClO}-E_{MgTiClO}$$

where $E_{Mg}$ denotes the energy per Mg atom in the bulk metal Mg. The $E_{TiClO}$ and $E_{MgTiClO}$ represent the total energies of pristine and magnesiated TiClO monolayers, respectively. The $E_{ads}$ for Mg on the site H_1_ (T_1_) is 1.22 (1.13) eV, while the adsorption of Mg on the site T_3_ possesses a higher $E_{ads}$ of 1.47 eV, indicating that the adsorption process between Mg atom and TiClO monolayer is an exothermic and spontaneous process.

In order to examine the electronic property of Mg-adsorbed TiClO, we calculate the PDOS of the TiClO monolayer for Mg adsorbed at the most favorable site, as depicted in Figure 4b. The PDOS structure shows that the metallic states of the TiClO monolayer are mainly contributed by Mg-*s* and Ti-*d* orbitals. Therefore, TiClO can maintain its metallic character after the adsorption of Mg, providing good electrical conduction during the charge/discharge process. Furthermore, we calculate the electron density difference to gain insight into the adsorption mechanism of the Mg atom on the surface of the TiClO monolayer. As shown in Figure 4b, we observe an apparent net loss of charges around the Mg atom and a net gain of charges in the intermediate area between Mg and O, indicating that electron transfer occurs from the Mg atom to TiClO. Based on the Hirshfeld charge analysis, the Mg atom donates about 0.46 electrons to the substrate. Clearly, the interaction between the Mg atom and TiClO monolayer is predominately ionic.

### 3.5. Mg Diffusion on Monolayer TiClO

**Figure 5:**
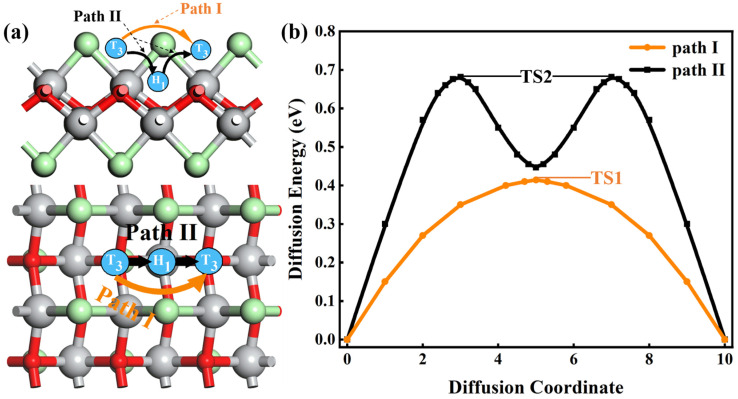
(**a**) Side and top views of the two possible migration paths of Mg on the TiClO monolayer. (**b**) The corresponding diffusion barrier profiles of two diffusion paths. TS1 and TS2 denote the transition states on paths I and II, respectively.

For an efficient anode of MIBs, the high mobility of the Mg ion is a key requirement that directly affects the rate performance. Therefore, we calculate the energy barriers of one Mg atom on the TiClO monolayer. Site T_3_ is the energetically most stable adsorption site, and site H_1_ is the second stable adsorption site. We investigate two typical diffusion pathways (path I and path II) between the nearest energetically favorable adsorption sites (Figure 5a), namely, T_3_ → T_3_ and T_3_ → H_1_ → T_3_. When the Mg atom diffuses along path I, there is only one peak point (TS1). While there are two peak points (TS2) when the Mg atom diffuses along path II, the TS positions are above of Ti atoms (Figure 5b). The Mg diffusion barriers along the path I (II) are about 0.41 (0.68) eV, which are higher than those of some typical 2D materials such as MnSb_2_S_4_ (0.32 eV) and TiS_3_ (0.29 eV) [43,44]. However, these results are much lower than those of BSi (0.86 eV) [45] and MoS_2_ (1.12 eV) [46], suggesting that Mg has tremendous diffusion capability along the armchair direction on the TiClO monolayer. The temperature-dependent diffusion constant (D) can be estimated by the Arrhenius equation [47]:

(3) $$D\approx exp(-\frac{E_{a}}{k_{B}T})$$

where $E_{a}$ and $k_{B}$ represent the energy barrier and Boltzmann constant, respectively. T is the environmental temperature. The Mg mobility along path I is about 10^4^ times faster than that along path II, showing the quasi-one-dimensional diffusion feature of TiClO.

### 3.6. Theoretical Specific Capacity and Open Circuit Voltage

**Figure 6:**
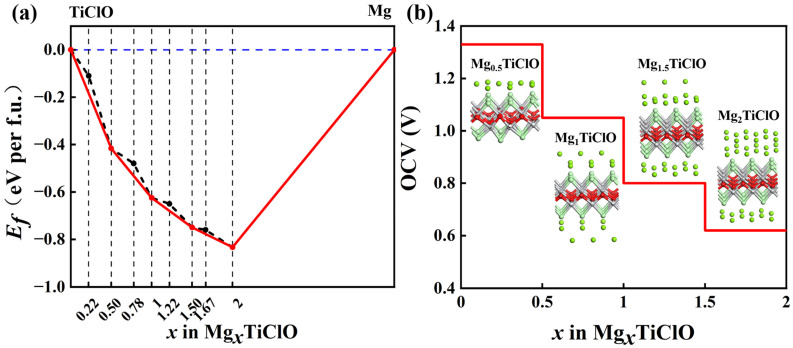
(**a**) Formation energies of Mg*_x_*TiClO systems, where the red solid line represents the convex hull and the black dotted line denotes the formation energies of Mg*_x_*TiClO. (**b**) The OCV profile of Mg on TiClO monolayer. The insets are the side views of the Mg-TiClO systems at different Mg concentrations.

The specific capacity is also a critical parameter for the practical application of ion batteries. We further investigate Mg atoms adsorbed on both sides of the TiClO monolayer and evaluate the theoretical maximum specific capacity with a 3 × 3 × 1 supercell (Ti_18_Cl_18_O_18_). Firstly, the Mg atoms are adsorbed above the most energetically favorable site (site T_3_), while the second adsorption layer is located on site H_1_. These two stable adsorption sites can totally accommodate up to 36 Mg atoms. Regrettably, for the third layer adsorption, the substrate cannot maintain its geometric configuration. Eight different Mg concentrations, Mg*_x_*TiClO (*x* = 0.22, 0.5, 0.78, 1, 1.22, 1.5, 1.67, and 2), are considered. To evaluate the relative stabilities of Mg*_x_*TiClO, we calculate the formation energy ($E_{f}$) of Mg-TiClO systems at each concentration by using the equation as follows:

(4) $$E_{f}={(E}_{{Mg}_{x}TiClO}-E_{TiClO}-xE_{Mg})/(x+1)$$

where $E_{{Mg}_{x}TiClO}$ and $E_{TiClO}$ are the total energies of the Mg*_x_*-TiClO system and the pristine TiClO monolayer, respectively. The *x* is the concentration of Mg atoms adsorbed in the TiClO monolayer. The $E_{Mg}$ indicates the total energy of an isolated Mg atom of bulk Mg metal. As illustrated in Figure 6a, four considered Mg adsorption conformers (Mg*_x_*TiClO, *x =* 0.5, 1, 1.5, and 2) lie on the solid line of the convex hull, suggesting that these structures are thermodynamically stable.

The maximum theoretical capacity of the TiClO monolayer can be obtained from the equation:

(5) $$C=z\times x_{max}\times F/M_{TiClO}$$

where z is the valance electron number (z equals 2 for Mg), $x_{max}$ represents the highest concentration of Mg ions, F is the Faraday constant (26801 mA h mol^−1^), and $M_{TiClO}$ denotes the molar mass of the TiClO monolayer. The computed theoretical specific capacity of TiClO monolayer reaches up to 1079 mA h g^−1^, which is superior to those of some reported 2D materials, such as C_2_N (588 mA h g^−1^) [48], Ti_2_C (687 mA h g^−1^) [49], VO_2_ (815 mA h g^−1^) [50], phosphorene (865 mA h g^−1^) [16], and TiS_2_ (957 mA h g^−1^) [51]. In addition, the lattice expansions of *a* and *b* for a fully magnesiated TiClO monolayer are 4.3% and 3.7%, respectively, suggesting its excellent cycling stability.

The open circuit voltage (OCV) is a determinative factor for high-performance MIBs. The OCV of Mg*_x_*TiClO is defined as follows:

(6) $$OCV=\frac{E\left({Mg}_{x_{1}}-substrate\right)+\left(x_{2}-x_{1}\right)E_{Mg}-E\left({Mg}_{x_{2}}-substrate\right)}{2(x_{2}-x_{1})e}$$

where $E\left({Mg}_{x_{1}}-substrate\right)$ and $E\left({Mg}_{x_{2}}-substrate\right)$ represent the total energies of ${Mg}_{x_{1}}TiClO$ and ${Mg}_{x_{2}}TiClO$, respectively. $E_{Mg}$ denotes the total energy of a Mg atom in the bulk metal. As depicted in Figure 6b, the calculated OCVs for Mg*_x_*TiClO are in the range of 0.62–1.33 V, with a numerical average OCV of 0.96 V, which are in the range of those of typical anode materials, such as graphite (~0.20 V) [52] and TiO_2_ (1.50 V) [22]. Both high specific capacities and suitable OCVs suggest that the TiClO monolayer has great potential for application in MIBs.

### 3.7. Mg Adsorption Property on Bilayer TiClO

**Figure 7:**
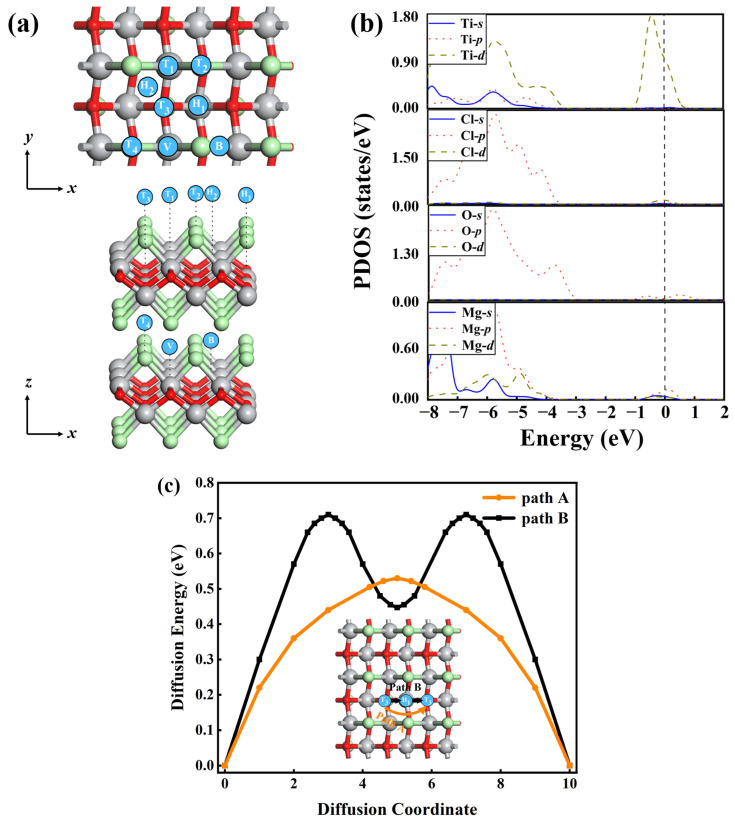
(**a**) Top and side views of the representative Mg adsorption sites on bilayer TiClO: top (T_1_, T_2_, T_3_, and T_4_), hollow (H_1_ and H_2_), bridge (B), and valley (V). (**b**) Partial DOS of Mg adsorption on bilayer TiClO at site T_3_. (**c**) The energy profiles of Mg diffusion along two migration pathways on the outside surface of bilayer TiClO.

Up until now, we have only explored the feasibility of Mg adsorption and diffusion on monolayer TiClO. Nevertheless, it is difficult to obtain a satisfactory electrode using the monolayer material in practical applications. Hence, we further evaluate whether the Mg atom could be adsorbed on bilayer TiClO. Two typical adsorption situations are considered: (1) Mg adsorption on the outer surface of bilayer TiClO; and (2) Mg insertion into the interlayer of bilayer TiClO. We perform the geometry optimization for the Mg-substrate systems by considering several possible adsorption sites (Figure 7a).

When the adsorption of Mg atoms happens on the surface of bilayer TiClO, sites T_3_ and H_1_ are the two most stable adsorption positions among all the considered sites, with the $E_{ads}$ of 1.76 and 1.62 eV, respectively. Clearly, the existence of a second TiClO layer enhances the interaction between Mg and substrate during the adsorption process. As for the Mg insertion into the interlayer of bilayer TiClO, the Mg atom prefers staying at site V with $E_{ads}$ of 1.45 eV. Clearly, the Mg atom is prone to adsorb on the outside surface rather than embed in the interlayer. Therefore, we can conclude that site T_3_ is the most favorable position for Mg on the bilayer TiClO as well as the TiClO monolayer.

In order to shed more light on the interaction between Mg and the outside surface of bilayer TiClO, we investigate the PDOS structure and Hirshfeld charge analysis. As illustrated in Figure 7b, the calculated PDOS clearly shows the metallic character of the Mg-bilayer TiClO system, which ensures excellent electronic conduction in the practical application of the electrode. According to PDOS analysis, the bands near the Fermi level are mainly originated from Ti-*d* and Mg-*p* orbitals, whereas the contribution from Cl-*d* and Mg-*s* states is limited. Moreover, the Hirshfeld charge analysis suggests charge transfer of ~0.55 electrons from Mg to bilayer TiClO at site T_3_, which is higher than that of TiClO monolayer and further demonstrates the enhancement of Mg binding strength in bilayer TiClO.

Then, we calculate the diffusion barriers on the outer surface of bilayer TiClO. Two different diffusion pathways are investigated, namely, path A (T_3_ → T_3_) and path B (T_3_ → H_1_ → T_3_), which are the same as those of the monolayer (Figure 7b). In the case of path A, the Mg atom moves directly between two neighboring sites T_3_ with a diffusion barrier of 0.53 eV. Nevertheless, the energy barrier along path B is 0.71 eV. The Mg mobility along path A is approximately 10^3^ times faster than that of path B, demonstrating the quasi-one-dimensional diffusion behavior of bilayer TiClO. In a word, the TiClO bilayer can remarkably enhance the Mg adsorption energy and maintain the quasi-one-dimensional diffusion feature.

### 3.8. Mg Adsorption Property on Trilayer TiClO

**Figure 8:**
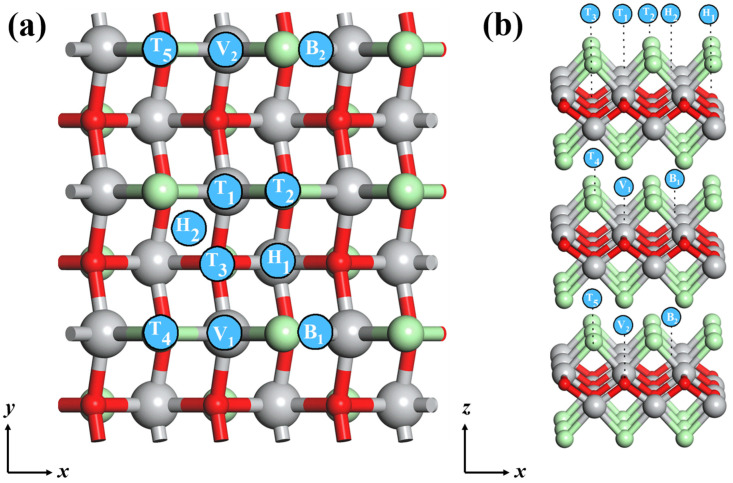

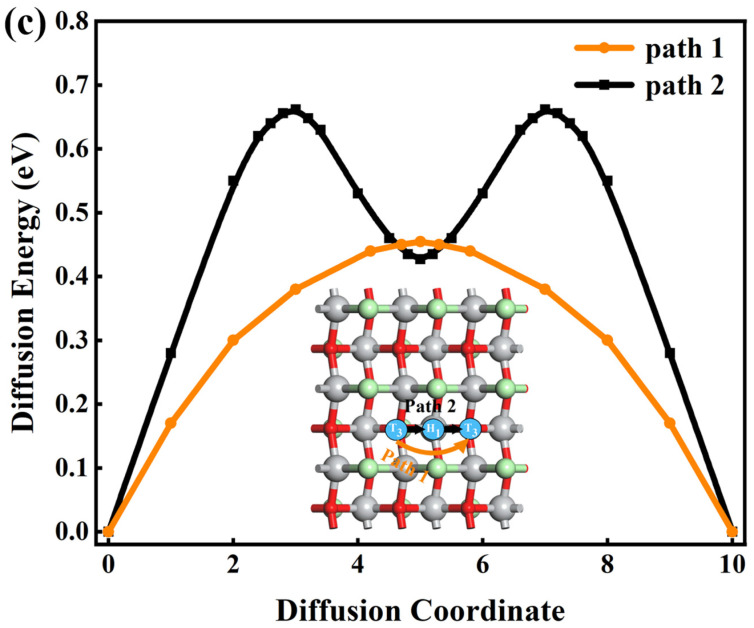
(**a**) Top and (**b**) side views of the considered Mg adsorption sites on trilayer TiClO: top (T_1_, T_2_, T_3_, T_4,_ and T_5_), hollow (H_1_ and H_2_), bridge (B_1_ and B_2_), and valley (V_1_ and V_2_). (**c**) The diffusion energy barriers of Mg diffusion along two migration pathways on the outside surface of trilayer TiClO.

In order to further explore the effect of a higher layer structure on Mg adsorption properties, we investigate the adsorption of Mg atoms on trilayer TiClO. Considering the symmetry of the three-layer structure, there are two types of regions for Mg adsorption. One region is on the outside surface of trilayer TiClO, and another region is in the interlayer of two neighboring layers of trilayer TiClO. Several possible adsorption sites are considered, as depicted in Figure 8a,b. After fully optimizing the geometry of Mg-TiClO systems, there are only two sites (site T_3_ and site H_1_) where the Mg atoms can be adsorbed. This phenomenon indicates that Mg atoms can only be adsorbed on the outside surface of trilayer TiClO. The computed adsorption energies of both sites are positive (1.59 eV for site T_3_, 1.36 eV for site H_1_), indicating the adsorption process between Mg and trilayer TiClO is spontaneous.

The energy barriers on the outside surface of trilayer TiClO are further calculated to evaluate the diffusion capacity of Mg atoms. Two diffusion pathways are considered according to symmetry of structure, as shown in Figure 8c. Path 1: T_3_ → T_3_; path 2: T_3_ → H_1_ → T_3_. The corresponding energy barriers are 0.45 (path 1) and 0.66 (path 2), which are close to the results of the TiClO monolayer. According to the Arrhenius equation, the mobility of Mg along path 1 is approximately 10^4^ times faster than that of path 2 at room temperature, indicating the quasi-one-dimensional diffusion character of trilayer TiClO. To conclude, the Mg adsorption properties of monolayer, bilayer, and trilayer TiClO, adsorption site, adsorption energy, diffusion energy barrier, number of electrons transferred, and electronic properties after adsorption are summarized and listed in Table 1.

## 4. Conclusions

By using first-principles calculations, we theoretically explore the potential feasibility of TiClO monolayer as an anode material for MIBs. Monolayer TiClO could be exfoliated from its bulk crystal with a moderate cleavage energy (1.13 J/m^2^). It possesses intrinsically metallic properties with excellent energetical, dynamical, mechanical, and thermal stabilities. From an electrochemical storage point of view, TiClO monolayer exhibits low diffusion barriers (0.41−0.68 eV), a suitable average electrode potential (0.96 V), and a high specific capacity (1079 mA h g^−1^). Importantly, the lattice expansion obtained for the fully magnesiated TiClO monolayer is small (<4.3%), demonstrating excellent cycling stability. Compared with pristine TiClO monolayers, bilayer and trilayer TiClO significantly enhance the Mg binding strength and maintain the quasi-one-dimensional diffusion feature. With the successful synthesis of some 2D Ti-based materials (e.g., TiOF, TiCl_2_) in recent years, our results could provide a reference for the reasonable design of promising anodes based on 2D Ti-based materials.

## Figures and Tables

**Table 1:** Adsorption properies of monolayer/bilayer/trilayer TiClO.

Structure of TiClO	Adsorption Site	Adsorption Energy (eV)	Diffusion Barrier (eV)	Eletrons Transfered	Electronic Property after Adsorption
monolayer	H_1_	1.22	0.41 (path I)	0.46 (site T_3_)	metallic
	T_1_	1.13	0.68 (path II)		
	T_3_	1.47			
bilayer	V	1.45	0.53 (path A)	0.55 (site T_3_)	metallic
	H_1_	1.62	0.71 (path B)		
	T_3_	1.76			
trilayer	H_1_	1.36	0.45 (path 1)		
	T_3_	1.59	0.66 (path 2)		

## Data Availability

The data in this study are available on request from the authors.
